# Effects of timing of initiation and planning on smoking cessation outcomes: study protocol for a randomised controlled trial

**DOI:** 10.1186/1471-2458-13-235

**Published:** 2013-03-18

**Authors:** Ron Borland, James Balmford, Elena Swift

**Affiliations:** 1 VicHealth Centre for Tobacco Control, The Cancer Council Victoria, 1 Rathdowne St Carlton, Melbourne, VIC 3053, Australia

**Keywords:** Randomised controlled trial, Smoking cessation, Planning

## Abstract

**Background:**

Recent theoretical and empirical work has led to debate over the benefit of delaying the implementation of a decision to quit smoking in order to plan the attempt. These two need not be linked, planning can occur before a commitment to quit is made, or after it is implemented, as well as in between. This study will test whether there are independent benefits for encouraging smokers to act immediately on a definite decision to quit smoking, and to engage in structured planning.

**Methods/design:**

A complex randomised controlled trial with a factorial design, testing the presence of a recommendation to quit immediately (or not) and encouragement to structured planning (or not) as additions to standard care, a web-based automated tailored advice program (QuitCoach). Participants are recruited from users of the QuitCoach who reside in Australia, do not report a mental health condition for which they are taking medication, are adult daily smokers, and at least open to the possibility of quitting. For the Immediate arm they could not have committed to quit within 2 days, while the Planning arm included all these and those quit within the last 4 days. This creates 6 groups: 2 × 3, with 2 × 2 fully randomised, and 2 only randomised for the planning arm. Follow-up assessments are conducted around 1 month (targeting two weeks after the quit attempt started), and 6 months later. The primary outcome is 6-month sustained abstinence at 6 months. Secondary outcomes include point-prevalence abstinence at both follow-ups, and making quit attempts during the intervention period. We will also explore differences in actual behaviour (timing and planning) by intervention, and relate this to outcomes.

**Discussion:**

This study will result in a better understanding of the roles of planning and delay in influencing the success of quit attempts.

**Trial registration:**

Australian New Zealand Clinical Trials Registry http://ACTRN12612000613808

## Background

Most attempts to quit smoking fail. Borland et al. [1] recently estimated that the average smoker makes around 1 unsuccessful quit attempt per year, defining attempts as quitting for at least a day. They found around 40% reporting attempts (average around 2 each), but that at least 20% of attempts are forgotten within the year. Smokers also report a similar number of aborted attempts (plans to quit that did not achieve 1 day of abstinence). This represents a lot of failed effort. Other researchers have estimated that only 3-5% of smokers are able to achieve prolonged abstinence for 6–12 months after a given unassisted quit attempt [2], an estimate consistent with the high levels of failure. Relapse is most common within the first week [2]. Good quality structured support and advice increases smoking cessation rates over those achieved in self-managed attempts, independent of any effect attributable to use of pharmacotherapy [3], but effects of both kinds of intervention are modest. However, evidence suggests little or no success in reducing relapse beyond the early days of an attempt [4,5]. There is a need for better strategies to reduce relapse.

It is widely believed that planning for a difficult task like quitting smoking should result in increased success. The importance of planning is emphasized in stage-based models of behaviour change [6], and is implicit in models that specify the factors leading to the formation of a behavioural intention, such as the Theory of Planned Behaviour [7]. Smoking cessation guidelines for health professionals e.g., [8-10] typically recommend that smokers be encouraged to set a quit date in the future (usually 1–2 weeks later), and to prepare for this date using a range of strategies including dealing with perceived barriers to quitting, seeking social support, considering the use of pharmacotherapy or behavioural assistance, keeping a smoking diary to better understand triggers to smoke, and developing coping strategies to deal with them.

Recently, theoretical and empirical developments have led to a questioning of the primary importance of planning for quit success. PRIME theory, a comprehensive theory of addiction proposed by West [11], has drawn attention to the instability of motivation [see also [12]; Borland, forthcoming] and to the primacy of impulses and emotions in the motivational system. According to the theory, smokers may experience ‘tension’ or dissonance about their smoking over a period of time without being moved to action, until a precipitating event occurs that triggers action. When a quit effort is triggered by such an ‘epiphany’, it may be launched with a motivational momentum that increases the likelihood of success. Delaying a quit attempt following such an event in order to plan for it may be detrimental, as it can lead to a decline in motivation over the planning period, thus resulting in the attempt being made on average during a period of reduced motivation. Related to this several recent studies, using retrospective reports, have found that those who successfully quit (typically stopped for more than 6 months) have been more likely to report that their quit attempt was spontaneous, i.e. occurred as soon as the decision was made, rather than allowing a period for planning, than those who reported a failed attempt [13-17]. A study of ours found much more complex patterns with some evidence of shorter planning periods associated with less success, but no effect for longer delays [18]. Population-based retrospective studies of cutting down to quit (a form of preparatory planning and something that precludes spontaneous quitting completely) find it also results in less success than abrupt cessation, even among attempts that result in a period of cessation [19].

At this point, it is premature to conclude that delay is detrimental. The Murray et al. [15] paper provided some evidence that at least some of the effects are due to differential forgetting. Forgetting could be because a lead-in period increases the duration and thus potential salience of the event for any given length of time quit, and does this proportionately more for short attempts. Alternatively, pre-quit periods might be disproportionately likely to be forgotten with time quit as they form a smaller part of the total event. However, if the effects are real, it suggests that some forms of preparatory activity are counterproductive. Borland’s (forthcoming) dual process theory of behaviour emphasises the continually changing, environmentally cued, reactive tendency to smoke, and argues that self-regulatory functions need to be able to sustain a level of motivation for change sufficiently long for both a quit attempt to be initiated and for it to become a stable new behaviour pattern. This requires effort, which is prone to be exhausted [20,21]. As effort is required in the preparatory stages, delay will bring forward the point of exhaustion, all other things being equal. So unless preparatory activity actually leads to the task becoming easier at a faster rate than the effort involved leads to exhaustion, relapse will be more likely under conditions of delay.

Up to around half of quit attempts are reported to start immediately the decision is made [13-18]. Cooper et al. [18] also found that a minority of attempts begin after a period of abstinence for other reasons (e.g. being too ill to smoke), with only around half delaying implementation. Qualitative research [15], an in-depth empirical study following smokers on a day by day basis [12], and our own work all point to greater complexity regarding what is meant by both spontaneity and planning. First, implicit in West’s model is that spontaneity relates to peaks in fluctuating levels of longer-term concern; that is, that ‘spontaneous’ quit attempts are typically preceded by periods of deliberation that are not strong enough to trigger action, rather than occurring completely out of the blue. Certainly, a lot more smokers report thoughts of quitting than go on to try in any given period of time [1]. Second, planning may be able to be carried forward from previous, especially recent attempts. Third, our work has found that a proportion of those reporting spontaneous quits also report cutting down to quit and/or using medications (e.g., Varenicline) that require a period of use prior to stopping smoking (unpublished data). Clearly these do not represent cases of spontaneously fully implementing a quit attempt.

There is clearly ambiguity as to what constitutes a spontaneous attempt and how that relates to planning. First, what has been caught up under ideas of spontaneity really covers two quite distinct concepts: the spontaneity of the decision to quit (i.e., whether it is made without any preparatory thought or related activity); and the immediacy of implementation (i.e., whether implementation occurs immediately the decision to quit is made or with some delay). Implementation can also either refer to stopping completely (full implementation), or beginning a process that will lead to full implementation; e.g., arranging for a doctor’s appointment to get prescription medication, beginning a period of preloading with medication, or starting a cut-down schedule. Where implementation is staged, the actual quit date might not be set until reaching the target window for quitting (e.g., for Varenicline from 1–2 weeks after initiating use), and implementation could then be ‘immediate’ or sometime in the future (delayed). This analysis suggests that it may be useful to distinguish four key concepts, defining a **spontaneous attempt** as one where the decision or process of deciding is initiated without any prior forethought (at least recent); **delay in initiation** as a gap between the decision to act and the initiation of the attempt; **initiation of implementation** as beginning to perform any action necessary to the chosen approach (e.g. obtaining medication); and **full implementation** as actually stopping smoking completely.

We now turn to a similar analysis of planning, or more correctly, pre-planning, as we are not considering on-the-spot planning to deal with actual challenges. The term ‘planning’ can refer to a wide range of possible activities from simply forming some intention to act (I am planning to quit), through to various forms of preparatory activity. The research referred to above has tended to assume that planning must occur before action, and that spontaneous attempts preclude the possibility of planning. It is true, where a spontaneous decision is fully implemented immediately, that there is no opportunity to pre-plan a quit attempt. Otherwise, there is the possibility of conditional planning (i.e. before deciding to quit) or of engaging in planning between making the decision and full implementation. However, while delay provides the opportunity to plan, it does not mean it will happen.

There are some aspects of planning that must by necessity occur before full implementation, such as deciding on how to quit (abruptly or by cutting down), whether to use help such as Varenicline that should start before actually quitting, and planning activities like keeping diaries of cigarettes smoked. However, many aspects of planning can equally occur before or after quitting, such as planning for high-risk situations and seeking social supports. Anticipating and forming strategies to deal with events that may precipitate relapse can occur after a quit attempt has started, though situations that will be encountered early on, such as dealing with strong situational cravings, would have to be prioritized. Assuming the person is not already craving a cigarette when they make the decision to quit, they should have some time to plan before any serious cravings occur, and except for the minority who experience almost continual cravings, should have time between bouts of craving to plan for future instances, even if they have to deal spontaneously with a couple of unplanned-for episodes of craving before their planning is complete. Certainly, the notion that the sequence must be to decide, plan, then implement is by no means a necessity, and what limited data we have suggests it is by no means the norm. There is also no good evidence as to whether the timing of these activities has any effect on their utility.

It is also plausible that the quality of planning is at least as important as its presence. Recent research has shown that a form of planning called an implementation intention can improve outcomes [22]. An implementation intention is a self-statement of the form when or if some specified event occurs, I will engage in some specified protective action; e.g., ‘when I get a craving to smoke while with my friend Jim, I will remind him that he has agreed to help me stay quit’. A meta-analysis of 94 studies found forming implementation intentions to have a medium-to-large effect on goal attainment, over and above the impact of forming relevant goal intentions [23]. More recently implementation intentions have been shown to facilitate ongoing goal striving (relapse prevention), protecting individuals from the influence of potentially disruptive inner states such as cravings [24]. It appears to be a new and important strategy for getting people to implement plans at the appropriate time. That said, all of the studies to date have either been of relatively straight-forward behaviours, or have only considered short term outcomes for more complex ones like smoking.

This study is designed to test (a) the potential benefit of structured support for planning, and (b) the potential benefit of recommending an immediate start to the implementation of a quit attempt.

Conducting RCTs of events where choice is under the control of smokers rather than researchers/clinicians is always complicated and often requires compromise. It is particularly problematic when smokers’ spontaneous choices might contribute to the therapeutic effect. We cannot randomize spontaneity, but independent of whatever pre-decisional planning smokers have engaged in, we can randomize to encouragement to quit at one time or another, and to the provision of structured help to facilitate planning, but are limited in our ability to constrain the timing of the planning that is undertaken.

Advising people to stop smoking immediately is unlikely to be a sensible intervention strategy because it precludes use of the forms of help that require a period of pre-quit use (e.g. Varenicline). Immediate implementation is defined here as immediately taking whichever ‘next step’ is required to ensure that the quit attempt takes place without avoidable delay, and only for some will this be stopping immediately. We will record instances of unavoidable delay, to see if this makes a difference.

In this study, we will recruit a group of smokers at a time when their motivation to quit is high, and at the point at which they seek cessation assistance, but who are not sufficiently advanced in their progress towards becoming an ex-smoker to preclude the provision of either or both interventions. The use of web-based cessation help is an ideal setting, as smokers can access it when considering quitting and there need be no delay. While some smokers access this form of help after quitting, most do so before, including many who have not yet committed to an attempt [25]. The QuitCoach [26], a demonstrably effective Internet-based automated tailored advice program developed by the authors [27], is designed to assess a smoker’s situation and provide immediate assistance. Users complete a 10-minute online assessment and receive tailored advice based on their answers, which can either be read on screen or downloaded as a PDF document. The program is designed to be used on multiple occasions, with return to the site recommended after a significant change has taken place, such as actually initiating a quit attempt, or to prevent relapse.

The primary hypotheses are that among smokers who seek help from QuitCoach, 6 month sustained abstinence will be greater among:

1. those encouraged to begin to implement a quit attempt immediately as compared with those supported to quit to their own timetable); and

2. those who are provided with a structured planning program with prompts to engage in planning activities and encouragement and supported to form implementation intentions as compared with those only provided motivational messages and general encouragement to plan.

Secondary aims are to:

1. test for any interaction between the two interventions, particularly to see if the two add value when combined;

2. test the hypothesis of a dose–response relationship for actual use of the planning resources and with other planning activity;

3. test whether more dependent smokers will be more likely to benefit from the planning intervention;

4. test whether smokers with a recent history of quit attempts and those who report at baseline that they have already engaged in planning will be less likely to benefit from the planning intervention; and

5. explore the relative benefits of the timing of planning (e.g., more before vs more after) on success.

## Methods/design

This trial has two levels of randomisation: first into the provision of structured planning tools (YES/NO), and then among that proportion of the sample who have not committed to an imminent attempt or already quit, randomisation into encouragement to quit as soon as possible (YES/NO) (see Table 1).

**Table 1:** Study design

		**Recommendation to begin implementation of quit attempt immediately**		
		**Randomised sub-group**		**Already committed**
		**Yes, begin immediately**	**No, follow own timetable**	**Too late to intervene**
Structured planning	**Yes**	IP	CP	XP
	**No**	IC	CC	XC

Code: I – Immediate implementation; P – structured planning; C – Control: standard QuitCoach; X - ineligible.

Eligibility for the Immediate arm is limited to those without a fixed quit date, or with a quit date two or more days away. The Structured Planning arm is open to all current smokers regardless of quit date, and also includes those quit for less than 4 days. The 2 × 2 design allows a test for interaction effects between the two aspects of each arm. The addition of the two supplementary conditions allows for a randomised test of the utility of structured planning on a broader sample, and non-randomised comparisons of the differential success rate of the QuitCoach as a function of participants’ progress toward quitting at recruitment (no quit date, quit dates of various times in the future, or already quit).

### Participant recruitment

Participants will be recruited from QuitCoach users. Eligibility will be limited to those who are over 18 years of age, who reside in Australia, do not report a condition that would require referral to an in-person service (i.e. medicated for a mental health condition), and are interested in quitting smoking. For the Immediate arm it excludes those committed to an attempt within the next 2 days, but these are included along with those quit for up to 3 days in the Structured Planning arm. Interest in quitting is assessed by: “Which of the following best describes your current thinking about quitting? (a) I am planning to quit; (b) I am just open to the possibility (both eligible); (c) I am not interested in quitting in the near future (not eligible)”. QuitCoach users who do not consent to participate in the research receive the standard program.

Following the questions to establish eligibility (all part of the standard QuitCoach assessment), eligible participants are invited into a study “to find out whether the way people use the QuitCoach affects their chances of successfully quitting”. They are also told the study involves completing two short follow-up surveys, and that they might get access to some added functionality of the QuitCoach which are not yet available to everybody. They are also told: “We ask you to consider whatever recommendation the QuitCoach makes carefully and to follow the advice, unless you have good reasons not to.” Those consenting are sent an email welcoming them to the study and the brief research description.

We will use anonymous group data to assess whether those who agree to participate in the research differ from other users of the QuitCoach who meet the eligibility criteria.

#### Randomisation and allocation concealment

Randomisation is via a hidden binary number generator in the on-line recruitment survey which is activated after the person consents, allocating a code of 0 or 1 for each arm of the study (i.e., 0,0 = neither immediate implementation nor structured planning; 0,1 = no immediate implementation, receiving structured planning; 1,0 = receiving recommendation to quit immediately but no structured planning; 1,1 = receiving both interventions). Participants know what they are receiving (as blinding of participants is impossible in trials of behavioural interventions), but they are not told about the alternative arms, so are blind to differences between what they and others are receiving.

#### Interventions

**Base condition** This is the standard QuitCoach (the CC arm in Table 1). The QuitCoach consists of an assessment phase where users are asked a range of questions tailored to their progress towards quitting. No advice is provided during the assessment, except to a subgroup not eligible for this trial who report a mental health condition for which they are taking medication, who are recommended early in the assessment to seek medical advice in conjunction with use. On completion of the assessment users are provided with a tailored letter that provides a mix of personalised advice and encouragement tailored to their quit progress, level of addiction, self-efficacy, smoking-related beliefs, social supports, base levels of positive and negative affect, and identified challenges in dealing with temptations to smoke. Users are encouraged to quit when they are ready. The advice provides suggestions about the use of planning strategies, but they are not particularly highlighted, nor is any direction as to how to implement them provided in the letter. Third, there is provision of a range of untailored resources, which the tailored letter refers to where relevant, and those referred to are highlighted as being of potential use. Users are encouraged to use the program multiple times, and on second and subsequent times, feedback is provided not only on reported states at that time, but on progress from the previous assessment. Email reminders are sent to encourage return. Based on the evaluation of an earlier version of the program, only around one quarter (27%) return for a second assessment [25], even though multiple visits is associated with better outcomes [27].

More information about QuitCoach is available in the supplementary materials accompanying Borland, Balmford and Benda [28]. Readers who would like to see how the QuitCoach differs for each study condition can do so at http://historical.quitcoach.org.au (instructions for accessing the content of each condition are provided on the site).

**The immediate implementation arm (IC and IP groups)** This involves a modified version of the standard QuitCoach with key elements of the intervention delivered during the assessment stage. Those in this arm are encouraged (recommended) to quit immediately, or as soon as practically possible, during their initial QuitCoach assessment (and any subsequent assessments, if they have not already quit) (‘We suggest you think about setting a quit date right now. There is nothing to be gained by delaying it’). They are provided with a brief explanation of the reasons for doing so: that some recent research suggests that quitting as soon as you decide may increase the likelihood of success over delaying implementation. Those who have already set a quit date are encouraged to bring their quit date forward (‘We suggest you bring your quit date forward, as there is nothing to be gained by delaying it. Choose the earliest time you can’), ideally quitting immediately, later today or first thing tomorrow morning. Others are encouraged to take the next step toward quitting as soon as possible (e.g. for those planning to take prescription medication ‘We recommend you call the doctor to make an appointment immediately’), implement their quit attempt immediately they have done all they need to do, and to do this preliminary work in as short a period as possible (e.g. for those planning to cut down, ‘if you want to cut down, take no more than 4 more days to quit completely’). This is all done during the assessment phase. In addition, any commitment to immediate implementation is reinforced in the advice letter, and for those not taking up the suggestion during the assessment, they are encouraged to implement as soon as they feel ready to commit.

**The structured planning intervention** The structured planning augmentation of the QuitCoach (the IP, CP and XP arms of the study) consists of additional highly prescriptive instructions for planning a quit attempt, organised around two main tools; a To Do List (TDL) and a Problem Planner (PP). The To Do List operates something like the Task function in some computer diaries. It prompts the user by listing the most important things they need to do to increase their chances of quitting smoking successfully, with an indication of when they need to be done. The Problem Planner provides a template for the development of implementation intentions to survive identified problem situations.

**Figure 1:**
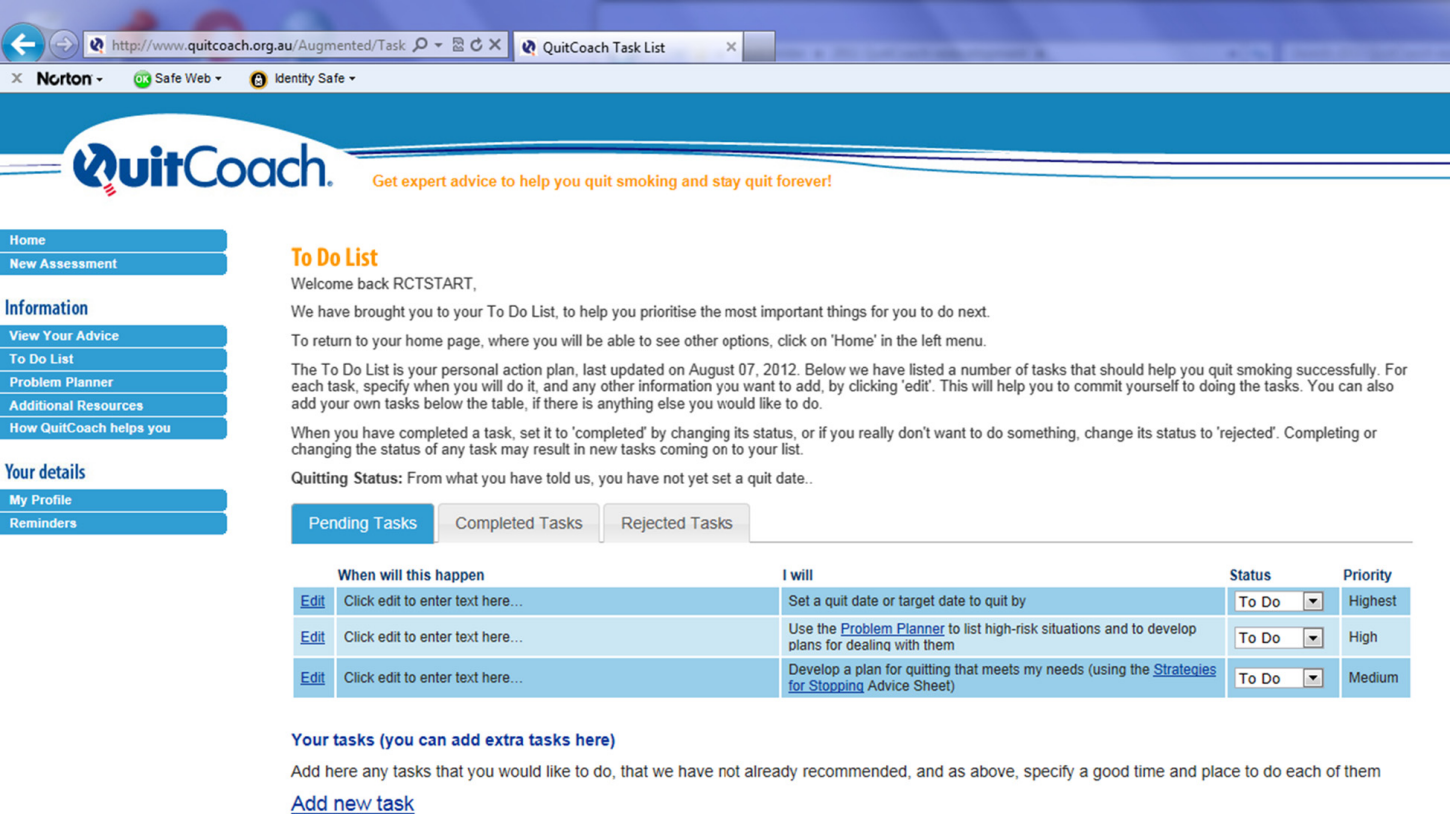
**Example screenshot of the to do list.** A screenshot of the TDL for a study participant, displayed upon their return to the QuitCoach.

Figure 1 shows a screenshot of the TDL for a study participant, displayed upon their return to the QuitCoach. The content of the TDL is dynamic, based primarily on smoking status/intention to quit, but also on other responses to the QuitCoach assessment including static variables such as age and gender, as well as things like levels of temptations and urges to smoke.

A maximum of 3 tasks are displayed on an individual’s TDL at any one time, with the list tailored both in terms of the tasks that appear on it, and the order in which they appear. The limit is to prevent the task seeming too overwhelming, and to maximise the completion of the tasks we consider most likely to be helpful. Each task is accompanied by a priority rating (highest, high or medium). Tasks potentially listed for inclusion on the TDL include: (a) finding out more about the health effects of smoking; (a) making or reviewing a list of your reasons for quitting; (b) deciding on a plan for how to quit, e.g. cold turkey or by systematically reducing consumption; (c) deciding on and, if relevant, implementing a plan for use of medication; (d) developing and documenting both cognitive and behavioural plans for coping in high-risk situations, using the Problem Planner; (e) making plans to ensure that friends and family are on-side to provide social support; (f) planning a schedule of rewards for achieving abstinence goals; (g) making your home smoke-free; (h) writing a list of things you will miss when you quit, and considering how these things can be achieved in other ways; (i) learning simple stress management techniques; (j) reviewing what went wrong on the previous quit attempt; (k) creating a smoking diary to better understand your smoking, and (l) if quit, spending a few minutes preparing for the day ahead. Many of these tasks refer participants to resources available on the QuitCoach site. These resources are available to all QuitCoach users, but only those in the structured planning arm will be directed to them in a systematic way. Participants may also add their own tasks to their TDL, but these do not displace existing tasks on the list.

Participants are encouraged to form implementation intentions for each listed task to help ensure each behavioural proscription or self-identified strategy for dealing with tempting situations is carried out. The instruction to do so is worded: ‘In the box below, write when you will do this, and anything else you want to add that will help you’. Tasks on the TDL are able to be marked as completed (at which point they are removed from the list and added to a ‘completed tasks’ list) or if the person decides not to do it, rejected (and added to a ‘rejected tasks’ list). Tasks marked as completed or rejected are replaced on the TDL by the next-highest priority task, if one exists.

The Problem Planner (PP) is a dynamically-generated form, presented in the form of a ‘wizard’ across several screens, into which participants can enter strategies for dealing with the situational temptations to smoke they identified during the QuitCoach assessment as likely to be difficult for them. For each identified strategy, participants are encouraged to form an implementation intention to help ensure that the strategy is remembered and implemented when needed (for example, ‘**If I** am at the pub and my friends go outside to smoke, **then I will** stay indoors and think about how much better it is not to smell of secondhand smoke). The PP wizard is structured into seven categories of tempting situations, of which five are presented to any one user. Three of them are mandatory (dealing with crises, strategies for slipups and other situations (those not covered earlier)). Two are chosen from the other four (coping with stress, social situations, first cigarette of the day, and filling in time), based on responses to the QuitCoach assessment. Under each category of situation, participants can create as many ‘if-then’ statements as they wish. Upon completion of the wizard, the PP is compiled into a form that can be viewed on screen and printed as a PDF document. On subsequent visits to the site this form can be edited, should the participant wish to add or delete problem statements. The planning intervention is designed to support those receiving it, and thus we would expect those users to return more often to the QuitCoach than other participants, although nothing mandates this.

**Integration of the two interventions (IP group)** Because the Structured Planning intervention is tailored to any level of immediacy in quitting, there is nothing unique about this group except that more will be doing their planning after the initiation of their quit attempt and more after full implementation (as will the XP group).

**Common elements** Frequency of use of the QuitCoach is at the participant’s discretion, regardless of which of the four possible study arms they have been randomised to. Thus, there is no set endpoint for delivery of the intervention. All participants will be reminded to return to the site via emails sent periodically over several weeks, scheduled according to their readiness to quit.

#### Assessment tools

We collect research information at three points: 1) at baseline, incorporated into the QuitCoach assessment (nearly all questions used are part of the standard assessment); 2) a first follow-up two weeks after the estimated quit date for each participant, or where the quit date is unknown or a quit date has not been set, a month after recruitment; and 3) A final follow-up 6 months after the identified quit date for each participant, or a comparable time after recruitment if no quit attempt was reported at the initial follow-up. Follow-up assessments will be conducted over the Internet, using email invitations including a web link to prime respondents to do the surveys online, or for those failing to respond to this, by telephone interview (the emailed invitation includes a notification that those failing to respond will be called).

The QuitCoach assessment, used as the baseline survey, includes validated measures of sociodemographics, quitting history, intention to quit [29], self-efficacy to quit [29], nicotine dependence [30], pros and cons of smoking and temptations to smoke [31], determination to quit [32] and a 5-item measure of positive and negative affect. All of these are used in the advice tailoring algorithms. To this we have added measures of rated quality of life, health status, perceived usefulness of planning, and the extent to which planning has already taken place on the current quit attempt (prior to recruitment). The base assessment will also ask for residential details (i.e., postcode) with which to determine socio-economic status as measured by SEIFA levels of area disadvantage [33].

At the first follow-up, we will assess smoking status, including implementation of the suggested quit date and actual quit date. We will ask about the extent of any planning undertaken since the baseline survey both pre- and post-full implementation of quit attempts (as relevant). We will measure use of other cessation support and use of pharmacotherapy (both pre-and post-implementation). The questions on affect will be re-asked in order to assess stability of mood. Finally, among those quit, we will measure self-efficacy to stay quit, extent of cravings [34], and determination to stay quit [32].

The final (6-month) follow-up will be restricted to measuring outcomes; smoking status, duration of time quit, and any lapses.

Engagement with the intervention will also be measured using naturally collected unobtrusive objective measures of program use [35], using web server log files and session identifiers linked to individual login names. Data collected will include the number of QuitCoach assessments completed and partially completed, number of log-ins to the site, number of openings of the advice page, To Do List and Problem Planner, number of edits of the To Do List, and number of times the additional resources page is viewed.

#### Sample size calculation

As noted earlier, we expect that a minority of participants will not comply with the recommendations to which they had been randomised. Based on levels of intervention usage achieved in a previous study in which participants were offered use of QuitCoach, a text messaging program or a combination [36], we anticipate at least 50% of those offered a program of structured planning will undertake a criterion level of planning (defined as one or more of seeking social support, practicing replacement strategies, or forming plans for coping with challenges, as reported at the first follow-up), whereas less than 5% of those in the IC and CC conditions will do so. We expect around 60% acceptance of the immediate implementation recommendation. This effectively means that the real effect of the intervention will be around twice the observed difference, which must be taken into account in power calculations.

We consider a 5% difference in absolute success rates (6 month sustained abstinence) to be highly significant. The expected base cessation rate for the control group (CC) is 18%, based on a comparable group from [28]. We propose a sample size of 600 per group (1200 per main effect comparison), which will provide 86% power to find a 5% increase in the success rate (23%) in the IC and CP conditions. We will have 98% power to find a 10% increase in the success rate (28%) in the IP condition over the CC condition, assuming no interaction. We have 80% power to find a difference of 6.6% between the IP and CC conditions [37]. A total sample size of 2400 across the 6 groups (i.e., with 800 per main effect comparison, with 800 in the XP and XC conditions combined) provides 68% power to find a 5% increase in the success rate in the IC and CP conditions.

#### Outcomes and data analysis

The primary outcome measure will be the percentage of smokers reporting 6-month sustained abstinence at the final follow-up, with supplementary analyses of the percent making a quit attempt assessed at 2-week post-implementation date, and point-prevalence abstinence at each follow-up point. The primary analyses will use intention to treat analysis with various strategies for dealing with missing data (all will be followed up regardless of engagement with the interventions). Some analyses will be conducted using only reported outcomes, especially those that involve possible mediators or moderators of effect, which will include logistic regressions. Biochemical validation of cessation outcome is considered unnecessary in larger population-based studies (particularly where there are low demands on the participants and no face-to-face therapist contact) as levels of misrepresentation are generally low and unsystematic [38]. This is an effectiveness trial where randomisation is to a set of recommendations which we know a significant proportion of participants will not follow. We will monitor both the choices smokers make around timing of implementation and the extent to which they comply with exercises and advice around structured planning as these should mediate outcomes see [39]. In doing so we will ask about any pre-decisional planning and prior quit experience. Nonetheless, the primary analysis will explore condition by outcome, independent of intervention compliance. Important secondary outcomes are whether the proportion of smokers who accept the offer to schedule their quit attempt and/or implement criterion levels of planning differs by condition, and the prospective relationship between those actions and success. We will also assess the impacts of the interventions and the outcomes on measures of quality of life, affect (particularly signs of depression), and overall health. This will be done both as a function of allocated condition and by achieved outcome.

#### Ethical review

The study protocol was approved by the Cancer Council Victoria Human Research Ethics Committee (HREC No. 1108).

## Discussion

This study will result in a better understanding of the roles of planning and delay in influencing quit attempts. It is the first large-scale population test of the utility of implementation intentions and if it shows they convey real benefits, should lead to their widespread adoption in cognitive behavioural interventions generally. It will also lead to advances in our understanding of how hard-to-change behaviours can be modified. This will allow us to provide advice both to smokers thinking of quitting, and to smoking cessation services, of the optimal way to configure quit attempts. We are also likely to have enhanced the capacity of QuitCoach in terms of increased efficacy and participation, thus increasing the potential impact of one component of Australia’s current suite of services for smoking cessation.

If smokers should be encouraged to quit spontaneously rather than delaying in order to plan, it has profound implications for the delivery of all cessation services. It would demand that help programs have the capacity to respond rapidly, rather than place people in queues to be managed on the service’s timeline.

## Competing interests

The authors declare that they have no competing interests.

## Authors’ contributions

RB and JB conceived of the study and drafted the manuscript. ES contributed to the implementation of the study and the drafting of the manuscript. All authors read and approved the final manuscript.

## Pre-publication history

The pre-publication history for this paper can be accessed here:

http://www.biomedcentral.com/1471-2458/13/235/prepub
